# A ceRNA network mediated by LINC00475 in papillary thyroid carcinoma

**DOI:** 10.1515/med-2021-0389

**Published:** 2021-12-06

**Authors:** Yarong Yang, Wenjuan Hua, Mei Zeng, Liling Yu, Baijun Zhang, Liming Wen

**Affiliations:** Department of Nuclear Medicine, Wuhan Fourth Hospital/Puai Hospital, Tongji Medical College, Huazhong University of Science and Technology, Wuhan 430022, Hubei, China; Department of Anesthesiology, Wuhan Fourth Hospital/Puai Hospital, Tongji Medical College, Huazhong University of Science and Technology, Wuhan 430022, Hubei, China

**Keywords:** papillary thyroid carcinoma, ZCCHC12, miR-376c-3p, LINC00475, competing endogenous RNA

## Abstract

Papillary thyroid carcinoma (PTC) is the most frequent histological type of differentiated thyroid carcinoma. Long noncoding RNAs (lncRNAs) have been widely reported to play a key role in human malignancies, and PTC is included. This study aimed to find out the functions and mechanism of lncRNA LINC00475 in PTC. LINC00475 was upregulated in PTC cells and was mainly located in the cytoplasm according to reverse-transcription polymerase chain reaction analyses and subcellular fractionation assays. As shown by cell counting kit-8 assays, ethynyl deoxyuridine incorporation assays, wound healing assays, and transwell assays, LINC00475 knockdown suppressed cell viability, proliferation, migration, and invasion. Mechanistically, LINC00475 upregulated the expression of messenger RNA zinc finger CCHC-type containing 12 (ZCCHC12) by binding to miR-376c-3p. ZCCHC12 was a direct target gene of miR-376c-3p in PTC cells. The relationship between miR-376c-3p and LINC00475 (or ZCCHC12) in PTC cells was probed by luciferase reporter assays, RNA pulldown assays, and RNA immunoprecipitation assays. In addition, both mRNA and protein levels of ZCCHC12 were downregulated due to miR-376c-3p overexpression or LINC00475 silencing. ZCCHC12 overexpression partially reversed the suppressive effect of LINC00475 knockdown on malignant behaviors of PTC cells. In conclusion, LINC00475 promotes PTC cell proliferation, migration, and invasion by upregulating ZCCHC12 via the interaction with miR-376c-3p.

## 1 Introduction

Papillary thyroid carcinoma (PTC) is the most common type of thyroid malignancy, accounting for more than 80% of thyroid cancer cases in the world [1,2]. PTC occurs in people of any age from the young to the elderly and is more prevalent in a female with a male-to-female ratio of 1:2.58 [3,4]. In recent years, with great advancements made in anticancer therapy, the prognosis of PTC patients has been significantly improved [5]. Nevertheless, the death rate of patients with thyroid carcinoma is still higher than that of patients with other endocrine tumors [6,7]. Moreover, the recurrence rate of PTC is also high (approximately 30%) [8]. Therefore, it is necessary to explore the underlying mechanisms of PTC to improve the prognosis of patients with PTC.

Long noncoding RNAs (lncRNAs) are transcripts with over 200 nucleotides in length that lack the protein-coding potential [9,10,11]. lncRNAs participate in the progression of cancer by regulating biological processes of cancer cells such as proliferation, epithelial–mesenchymal transition, and stem cell functions [12,13]. Many lncRNAs were reported to play an essential role in the development of PTC. For example, lncRNA LOC100129940-N was discovered to be upregulated in PTC and promote cell invasion and tumor progression [14]. Yuan et al. found that lncRNA HOXA distal transcript antisense RNA (HOTTIP) modulates miR-637 expression to regulate PTC cell proliferation, migration, and invasion [15]. Furthermore, lncRNAs can function as competing endogenous RNAs (ceRNAs) for microRNAs (miRNAs) to regulate the expression of downstream messenger RNAs that are direct target genes of miRNAs [4,16,17]. A considerable number of lncRNAs have been revealed to take part in PTC progression [18]. For example, Zhang et al. discovered that lncRNA nuclear paraspeckle assembly transcript 1 promotes PTC progression by upregulating the expression of kallikrein-related peptidase 7 via interaction with miR-129-5p as a ceRNA [19]. lncRNA small nuclear RNA host gene 3 facilitates PTC cell migration and invasion *in vitro* by acting as a ceRNA against miR-214-3p to modulate the expression of proteasome 26S subunit, non-ATPase 10 [20]. Based on bioinformatics analysis and our experiments, the functions and mechanism of LINC00475 in PTC were investigated. Previously, the comprehensive analysis revealed that LINC00475 can mediate the ceRNA network in renal carcinoma [21]. The involvement of LINC00475 in PTC has not been studied yet. Herein, LINC00475 was found to interact with miR-376c-3p to elevate the expression of zinc finger CCHC-type containing 12 (ZCCHC12).

ZCCHC12, also known as Smad-interacting zinc finger protein 1, is an oncogene in PTC [22]. According to previous studies, ZCCHC12 is upregulated in PTC and is a potential molecular marker of PTC [23]; ZCCHC12 downregulation suppresses PTC cell proliferation, migration, and invasion [22]. Moreover, bioinformatics analysis also revealed high ZCCHC12 expression in thyroid carcinoma tissue samples and normal tissue samples. However, the underlying mechanism of ZCCHC12 in PTC has not been reported yet.

We explored the underlying molecular mechanism of ZCCHC12 and how LINC00475 was involved in PTC progression for the first time. Results from this study may indicate that LINC00475 is a potential target for the diagnosis and therapy of PTC, which may help to achieve a better prognosis of PTC patients.

## 2 Materials and methods

### 2.1 Cell lines

PTC cell lines (8505C, TPC1, and SW1736) and human normal thyroid epithelial cell line (Nthy-ori 3-1) were purchased from the Chinese Academy of Sciences (Beijing, China). Another PTC cell line (BCPAP) was obtained from DSMZ (Braunschh, Germany). These cells were cultured in Dulbecco’s Modified Eagle Medium (DMEM; Gibco, USA) added with 10% fetal bovine serum (FBS; Gibco, USA) and 1% streptomycin (Gibco). Cells were cultured in a humidified atmosphere at 37°C containing 5% CO_2_.

### 2.2 Cell transfection

Short hairpin RNAs targeting LINC00475 (sh-LINC00475#1/2) and the negative control short hairpin RNA (sh-NC) were purchased from GenePharma (Shanghai, China). pcDNA3.1/ZCCHC12 was used to overexpress ZCCHC12 with empty pcDNA3.1 as the control (GenePharma). miR-376c-3p mimics and its negative control NC mimics (RiboBio, Guangzhou, China) were used for miR-376c-3p overexpression. Cell transfection was conducted using Lipofectamine 3000 (Invitrogen, Carlsbad, CA, USA) following the manufacturer’s protocols (Table 1).

**Table 1:** Sequences of shRNAs for cell transfection

shRNA	Sense (5′-3′)	Anti-sense (5′-3′)
sh-LINC00475#1	GCTTATTGCTGTTAGTTTA	TAAACTAACAGCAATAAGC
sh-LINC00475#2	GGACTTGTTTGAAAGGTTA	TAACCTTTCAAACAAGTCC
sh-NC	GTGTTTTTATTAAGCCTTG	CAAGGCTTAATAAAAACAC
sh-ZCCHC12#1	GAGCTTCTATGTCAATAAA	TTTATTGACATAGAAGCTC
sh-ZCCHC12#2	GGTATAATTTATTGTTAAA	TTTAACAATAAATTATACC
sh-NC	TTAGCAGTTATAACAGTAC	GTACTGTTATAACTGCTAA

### 2.3 Reverse-transcription polymerase chain reaction (RT-qPCR)

RT-qPCR was performed for the detection of levels of lncRNAs, miRNAs, and ZCCHC12. Extraction of total RNAs was performed using TRIzol reagent (Sigma-Aldrich, St. Louis, MO, USA). Reverse transcription was conducted by Super M-MLV reverse transcriptase (Promega, Madison, WI, USA). RT-qPCR analysis was carried out with SYBR Green (Sigma, USA). Expression levels of lncRNAs and ZCCHC12 were normalized to the expression of glyceraldehyde-3-phosphate dehydrogenase (GAPDH) while U6 was used as an endogenous control for miRNA expression. We calculated the relative expression using the 2^−ΔΔCt^ method. Sequences of primers used in RT-qPCR are provided in Table 2.

**Table 2:** Sequences of primers used for reverse transcription PCR

Gene	Sequence (5′ → 3′)
ZCCHC12 forward	CTTGTTTCAGAGTTCGTCCC
ZCCHC12 reverse	TAGGACCGAGACTTCTCCC
miR-376c-3p forward	AACATAGAGGAAATTCCACG
miR-376c-3p reverse	GCAGACAGCCGAGTACATCTT
miR-325-3p forward	GCCGAGCTATCCTCCACGAG
miR-325-3p reverse	CAGTGCGTGTCGTGGAGT
LINC00475 forward	CTCCTGAAGATACAGCAGC
LINC00475 reverse	GGGTGGAGTTATAGACATGC
GAPDH forward	TCATTTCCTGGTATGACAACGA
GAPDH reverse	GTCTTACTCCTTGGAGGCC
U6 forward	GGATCAATACAGAGCAGATAAGC
U6 reverse	CTTTCTGAATTTGCGTGCC

### 2.4 Subcellular fractionation assay

The localization of ZCCHC12 and LINC00475 in TPC1 and BCPAP cells was determined by subcellular fractionation assays. In this assay, the nuclear fraction was separated from cytoplasm using PARIS Kit (Life Technologies, Carlsbad, California, USA) according to the manufacturer’s instructions. Cells were incubated with lysis solution on ice for 10 min followed by 3 min of centrifugation at 12,000*g*. Cytoplasmic RNA was extracted from the supernatant, whereas the nuclear pellet was used to extract nuclear RNA. GAPDH was an endogenous control for cytoplasm, whereas U6 was that for the nucleus. LINC00475, ZCCHC12, GAPDH, and U6 expression levels in cytoplasmic and nuclear fractions of BCPAP and TPC1 cells were detected by RT-qPCR.

### 2.5 Cell counting kit-8 (CCK-8) assay

Cell viability was evaluated by conducting the CCK-8 assay (MedChemExpress, NJ, USA). Cells were plated onto 96-well plates (1 × 10^3^ cells per well). Then, CCK-8 reagent (MedChemExpress) was added to the culture medium after transfection for 24, 48, and 72 h, respectively. After another 4 h of incubation at 37°C, cell viability was detected by the optical density at 450 nm using the Universal Microplate Reader (SpectraMax i3; Molecular Devices, San Jose, CA, USA).

### 2.6 Ethynyl deoxyuridine (EdU) incorporation assay

Cell proliferation was assessed by the EdU assay. The replication of DNA was measured by an EdU labeling/detection kit (Thermo Fisher Scientific, Waltham, MA, USA). TPC1 and BCPAP cells were seeded into 96-well plates (5 × 10^3^ cells per well). After transfection for 48 h, 50 µM of EdU kit was added to the plates. Then, the mixture was incubated at 37°C under 5% CO_2_ for 2 h. Finally, the EdU-positive cells were observed under a fluorescence microscope (Olympus, Tokyo, Japan).

### 2.7 Wound healing assay

Wound healing assays were applied to evaluate PTC cell migration. After designated treatment, TPC1 and BCPAP cells were seeded into 6-well plates (2 × 10^5^ cells per well) and grown up to a monolayer. Then, the cell monolayer was scratched with a 200 µL pipette tip, and floating cells were removed by washing three times with phosphate-buffered saline (Thermo Fisher). Subsequently, at 0 and 24 h, cells were imaged with a phase-contrast microscope (Zeiss, Primovert, Axiocam). Finally, we quantified the migration of PTC cells with ImageJ Software (NIH; Bethesda, MA, USA).

### 2.8 Transwell assay

Cell invasion was measured by 24-well transwell chambers (Corning, Life Sciences, USA), and the upper chamber was precoated with Matrigel. Then, the cell suspension was prepared using 200 µL serum-free DMEM and was added to the upper chamber. Additionally, 500 µL DMEM with 20% serum was supplemented to the lower chamber. After incubation for 48 h, cells on the lower surface were stained with crystal violet (Sigma-Aldrich). Finally, a microscope (Olympus) was used to analyze the number of invaded cells.

### 2.9 Luciferase reporter assay

Luciferase reporter assays were used to confirm the binding between miR-376c-3p and ZCCHC12 (or LINC00475). Wild-type sequence of ZCCHC12 and mutant sequence of ZCCHC12 containing binding site with miR-376c-3p were subcloned into pmirGLO vectors (Promega) to construct ZCCHC12-Wt/Mut. The vectors were then cotransfected with NC mimics or miR-376c-3p mimics into BCPAP and TPC1 cells using Lipofectamine 3000 (Invitrogen) in accordance with the manufacturer’s protocol. In addition, the miR-376c-3p sequence was mutated and subcloned into pmirGLO vectors to establish miR-376c-3p-Mut, whereas the wild sequence of miR-376c-3p was used to construct miR-376c-3p-Wt. Next, miR-376c-3p-Wt/Mut was transfected into TPC1 and BCPAP cells with sh-LINC00475#2 or sh-NC using Lipofectamine 3000 (Invitrogen). Cells transfection was performed for 48 h. Finally, the luciferase activity was evaluated using a dual-luciferase reporter kit (Promega).

### 2.10 RNA pulldown assay

RNA pulldown assay was conducted to testify the binding between ZCCHC12 and miR-376c-3p. Biotinylated wild-type miR-376c-3p (bio-miR-376c-3p WT), mutant miR-376c-3p (bio-miR-376c-3p MUT), and negative control (bio-NC) were purchased from RiboBio (Guangzhou, Guangdong, China) and transfected to TPC1 and BCPAP cells. After transfection for 48 h, cells (1 × 10^7^) were dissolved in the lysis buffer and 80 U/mL RNasin (Promega). Next, the cell lysates were collected and cultured with Dynabeads M-280 Streptavidin (Invitrogen) for 3 h at 4°C according to the manufacturer’s instructions. Then, beads were washed six times in the lysis buffer. The retrieved supernatant was examined by RT-qPCR.

### 2.11 Western blot

We performed a western blot to measure the protein level of ZCCHC12. Radio-immunoprecipitation assay lysis buffer (Sigma-Aldrich, Darmstadt, Germany) containing phenylmethylsulfonyl fluoride was used for total protein extraction. A BCA protein assay kit (BOSTER, USA) was used to determine the protein concentration. Then, the protein was separated using 10% polyacrylamide gels and transferred to polyvinylidene fluoride (PVDF) membranes (Millipore, Billerica, MA, USA), which were then blocked with 10% skim milk for 1 h at room temperature. Afterward, the membranes were incubated overnight at 4°C with primary antibodies as follows: anti-GAPDH (ab8245; 1:1,000; Abcam, Cambridge, MA, USA), anti-ZCCHC12 (PA5-114001; Invitrogen). The membranes were treated with secondary antibodies (Abcam) for 2 h in the following day. Enhanced chemiluminescence (ECL; Pierce, Rockford, IL, USA) was used to visualize the band signals after exposure to a ChemiDoc™ XRSC system (Bio-Rad, CA, USA).

### 2.12 RNA immunoprecipitation (RIP) assay

RIP assays were conducted to detect the relationship between ZCCHC12, miR-376c-3p, and LINC00475. Cell lysates were incubated with protein magnetic beads (Thermo Fisher), which were conjugated with Ago2 and IgG antibodies overnight at 4°C. RIP assays were performed using the Magna RNA-Binding Protein Immunoprecipitation Kit (Millipore). The RNA immunoprecipitation was purified and detected by RT-qPCR.

### 2.13 Statistical analysis

We analyzed all statistics involved using GraphPad Prism 5 software. Data are demonstrated as the mean ± standard deviation. Two-tail Student’s *t*-test was used to compare differences between two groups, and one-way analysis of variance (ANOVA) with equal variance followed by Tukey’s *post hoc* analysis was used to analyze the differences among multiple groups. A value of *p* < 0.05 was considered as statistically significant.

## 3 Results

### 3.1 ZCCHC12 is highly expressed in PTC cells

**Figure 1:**
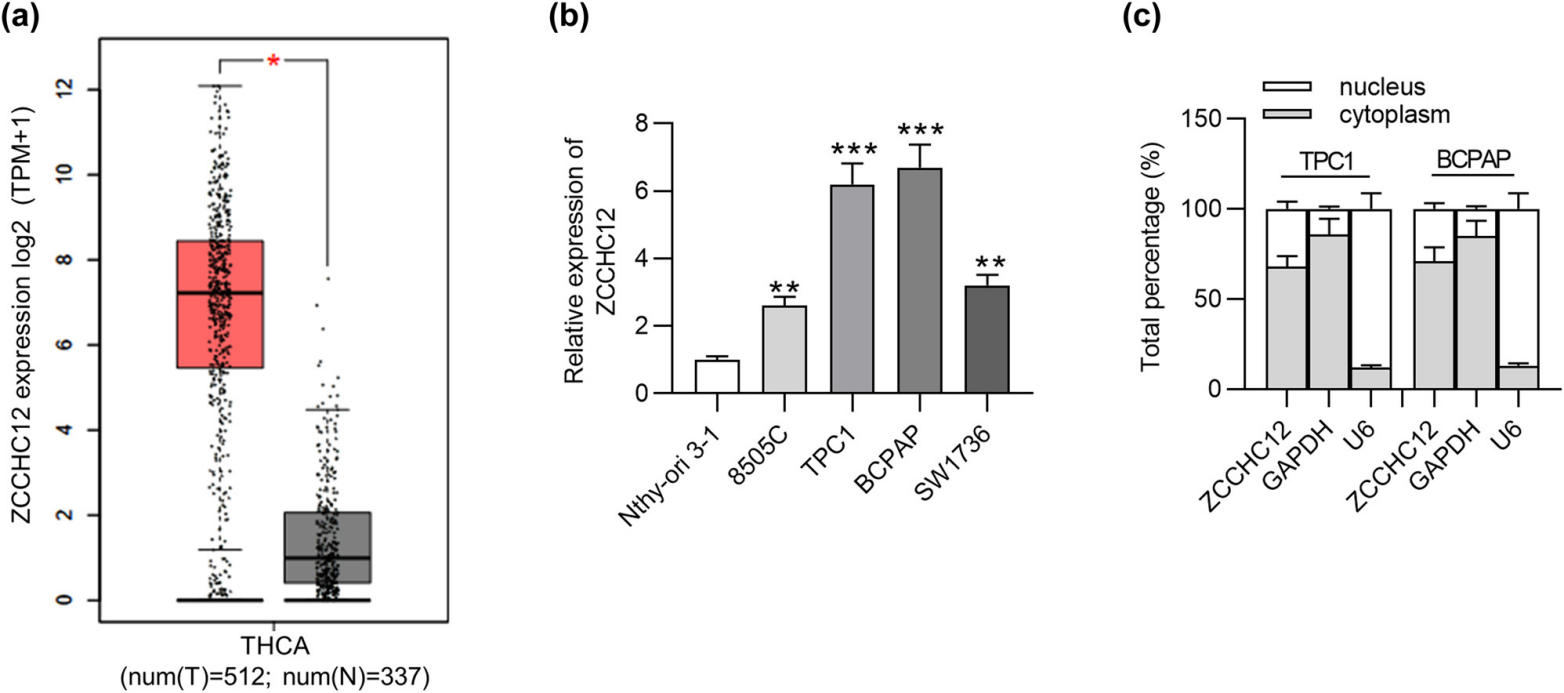
ZCCHC12 is highly expressed in PTC cells. (a) The expression of ZCCHC12 in thyroid carcinoma (THCA) tissues and normal tissues was analyzed by the GEPIA website. (b) RT-qPCR was conducted to assess ZCCHC12 expression in PTC cell lines (8505C, TPC1, BCPAP, and SW1736) and thyroid epithelial cell line Nthy-ori 3-1. (c) The localization of ZCCHC12 in TPC1 and BCPAP cells was detected by subcellular fractionation assays. *p < 0.05, **p < 0.01, ***p < 0.001.

GEPIA (http://gepia.cancer-pku.cn/) shows that ZCCHC12 expression is upregulated in tissue samples of thyroid carcinoma (*n* = 512) compared with that in normal tissues (*n* = 337) (Figure 1a). To investigate whether ZCCHC12 is dysregulated in PTC cells, RT-qPCR was performed. We found that ZCCHC12 expression was higher in PTC cell lines (8505C, TPC1, BCPAP, and SW1736), especially in TPC1 and BCPAP cell lines, than in thyroid epithelial cell line (Nthy-ori 3-1) (Figure 1b). Thus, TPC1 and BCPAP cell lines were used to conduct subsequent experiments. Subcellular fractionation assays suggested that ZCCHC was largely localized in the cytoplasm of TPC1 and BCPAP cells (Figure 1c). The results implied that ZCCHC12 functions at the posttranscriptional level. In summary, ZCCHC12 exhibited high expression in PTC cells and is primarily distributed in the cytoplasm.

### 3.2 ZCCHC12 knockdown inhibits PTC cell viability, proliferation, migration, and invasion

**Figure 2:**
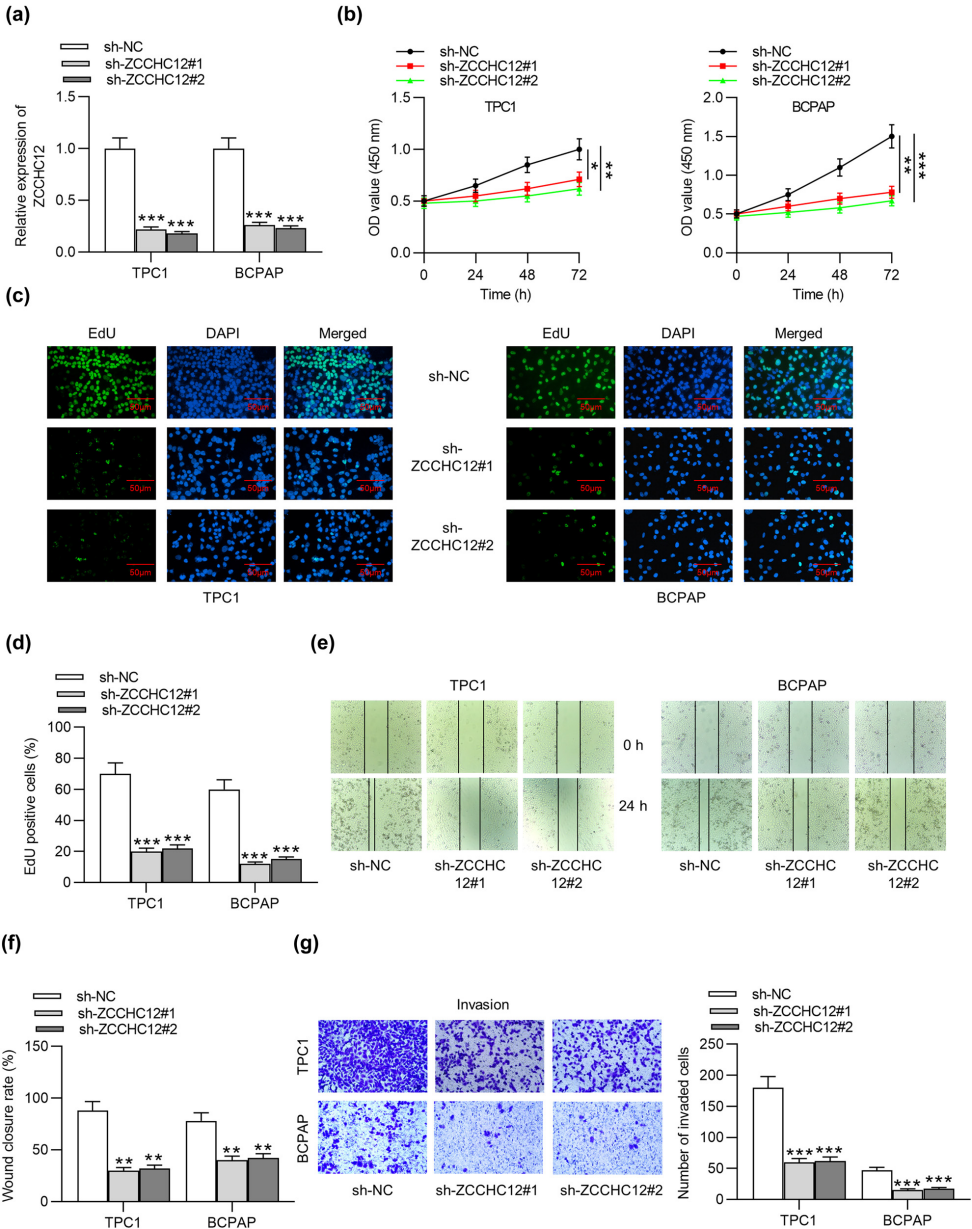
ZCCHC12 knockdown inhibits PTC cell viability, proliferation, migration, and invasion. (a) The expression of ZCCHC12 in TPC1 and BCPAP cells after transfection of sh-ZCCHC12#1/2 was detected by RT-qPCR. (b–g) After the transfection of sh-ZCCHC12#1/2, cell viability was assessed by CCK-8 assays, cell proliferation was evaluated by EdU assays, cell migration was measured by wound healing assays, and cell invasion was detected by transwell assays. *p < 0.05, **p < 0.01, ***p < 0.001.

Due to the high ZCCHC12 expression in PTC cells, loss-of-function experiments were conducted to explore the effect of ZCCHC12 on the cellular activities of PTC. First, RT-qPCR demonstrated that the ZCCHC12 level in TPC1 and BCPAP cells was significantly reduced after transfection of sh-ZCCHC12#1/2 (Figure 2a). Afterward, CCK-8 assays implied that the knockdown of ZCCHC12 suppressed the viability of PTC cells (Figure 2b). Additionally, the number of EdU-positive cells was reduced by ZCCHC12 depletion (Figure 2c and d). The finding implied that silenced ZCCHC12 inhibited PTC cell proliferation. Moreover, the wound closure rate of TPC1 and BCPAP cells was suppressed due to ZCCHC12 deficiency according to wound healing assays (Figure 2e and f). Transwell assays also suggested the decrease in the invasive ability of PTC cells mediated by silenced ZCCHC12 (Figure 2g). In conclusion, silenced ZCCHC12 represses PTC cell viability, proliferation, migration, and invasion.

### 3.3 ZCCHC12 is a target gene of miR-376c-3p in PTC cells

**Figure 3:**
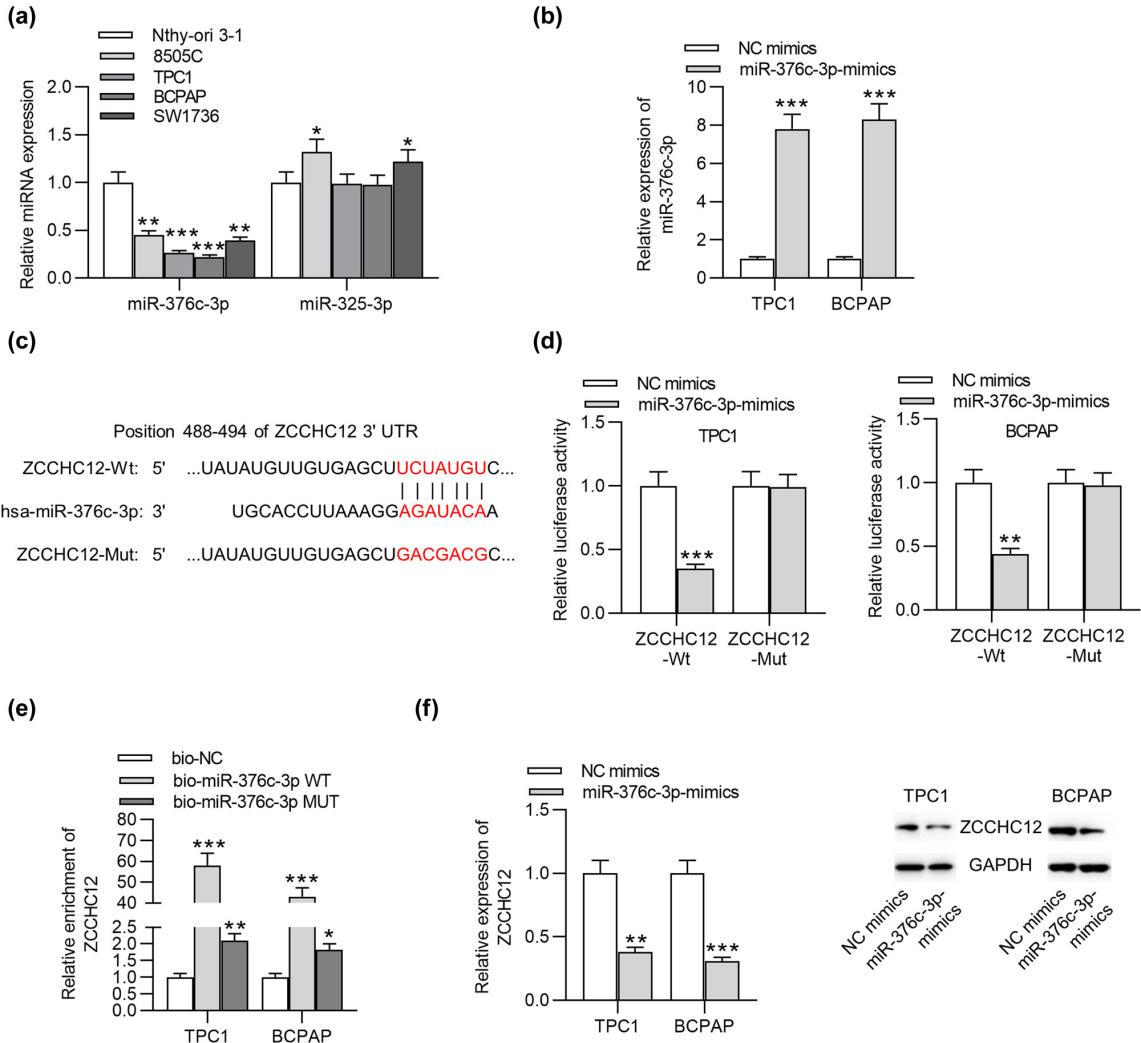
ZCCHC12 is a target gene of miR-376c-3p in PTC cells. (a) Expression levels of candidate miRNAs (miR-376c-3p and miR-325-3p) in thyroid epithelial cell line and PTC cell lines were examined using RT-qPCR. (b) RT-qPCR was performed to detect miR-376c-3p level in PTC cells with transfection of miR-376c-3p mimics. (c) The binding site between miR-376c-3p and ZCCHC12 was searched on the TargetScan website. (d and e) Luciferase reporter assays and RNA pulldown assays were conducted to testify the interaction between miR-376c-3p and ZCCHC12. (f) The influence of miR-376c-3p mimics on mRNA and protein levels of ZCCHC12 were evaluated by RT-qPCR and western blot. *p < 0.05, **p < 0.01, ***p < 0.001.

According to the ceRNA hypothesis, ZCCHC12 is involved in the lncRNA-miRNA-ZCCHC12 network. Specifically, 3′-untranslated region of ZCCHC12 was targeted by a miRNA, and a certain lncRNA positively regulates the expression of ZCCHC12 by interacting with the miRNA to reverse the inhibitory effect of the miRNA on ZCCHC12. Therefore, we searched for miRNAs that can bind to ZCCHC12 using TargetScan (http://www.targetscan.org/vert_72/), and two miRNAs (miR-376c-3p and miR-325-3p) possessing binding site with ZCCHC12 were identified. RT-qPCR revealed that miR-376c-3p showed significantly lower expression levels in TPC1 and BCPAP cells than in Nthy-ori 3-1 cells, whereas miR-325-3p expression showed little alteration in PTC cells compared with that in the control cells (Figure 3a). Next, results from RT-qPCR showed that miR-376c-3p levels were successfully elevated by miR-376c-3p-mimics in TPC1 and BCPAP cells (Figure 3b). TargetScan was utilized to predict the binding site between ZCCHC12 and miR-376c-3p, and a mutated ZCCHC12 fragment was also provided (Figure 3c). Luciferase reporter assays demonstrated that after co-transfection of miR-376c-3p mimics and ZCCHC12-Wt/Mut, the luciferase activity of ZCCHC12-Wt was significantly reduced and that of ZCCHC-Mut was not significantly affected in PTC cells (Figure 3d and e). As presented by RNA pulldown assays, ZCCHC12 showed high enrichment in the bio-miR-376c-3p WT group whereas no significant enrichment of ZZCCHC12 was found in bio-miR-376c-3p MUT (Figure 3e). The results further verified the interaction between miR-376c-3p and ZCCHC12. In addition, upregulation of miR-376c-3p repressed mRNA expression and protein levels of ZCCHC12 in PTC cells (Figure 3f). Overall, ZCCHC12 is a target gene of miR-376c-3p in PTC cells.

### 3.4 LINC00475 upregulates ZCCHC12 expression by binding to miR-376c-3p

**Figure 4:**
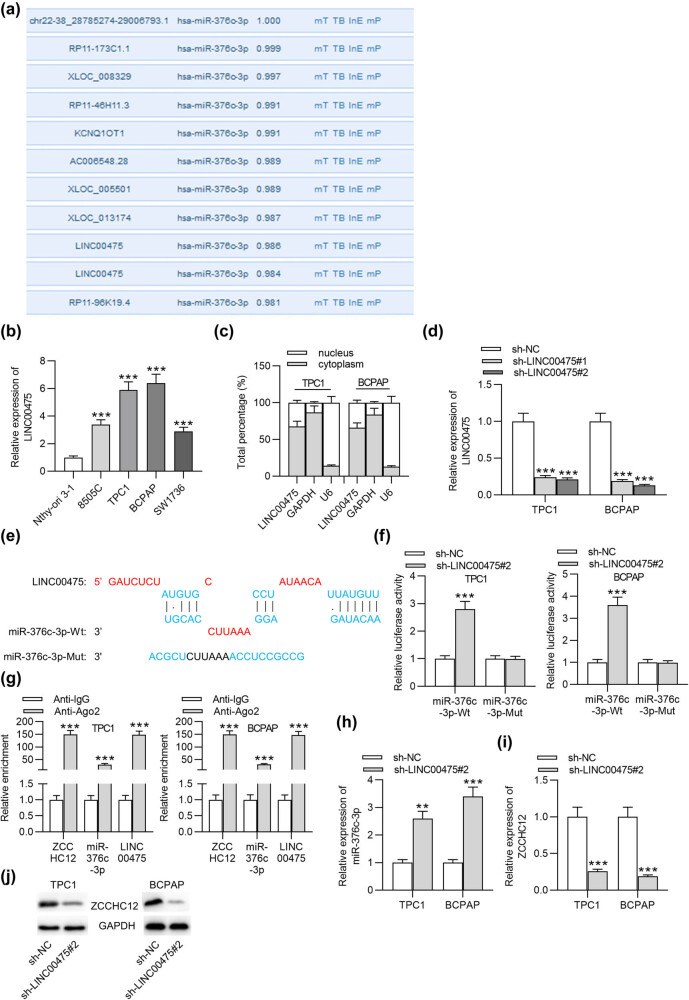
LINC00475 upregulates ZCCHC12 expression by binding to miR-376c-3p. (a) lncRNAs that can bind to miR-376c-3p were predicted with the LncBase. (b) RT-qPCR was conducted to detect the LINC00475 level in PTC cells and normal thyroid epithelial cells. (c) Subcellular fractionation assays were performed to determine the distribution of LINC00475 in TPC1 and BCPAP cells. (d) After transfection with sh-LINC00475#1/2, RT-qPCR was used for the detection of LINC00475 expression in PTC cells. (e) The binding site between LINC00475 and miR-376c-3p was predicted by the LncBase. (f) Luciferase reporter assays were conducted to testify the binding between LINC00475 and miR-376c-3p. (g) The interaction between miR-376c-3p and LINC00475 (or ZCCHC12) was further evaluated by RIP assays. (h) RT-qPCR was utilized to detect miR-376c-3p expression in PTC cells after transfection of sh-LINC00475#2. (i and j) RT-qPCR and western blot were conducted to assess ZCCHC12 expression in PTC cells after transfection of sh-LINC00475#2. **p < 0.01, ***p < 0.001.

Subsequently, the upstream lncRNA of the miR-376c-3p/ZCCHC12 axis was examined. According to the prediction of LncBase (http://carolina.imis.athena-innovation.gr/diana_tools/web/index.php?r=lncbasev2/index-predicted), we found 10 lncRNAs that can bind to miR-376c-3p, and LINC00475 was finally identified as it contains more than one binding site for miR-376c-3p (Figure 4a). RT-qPCR showed that LINC00475 was upregulated in PTC cell lines compared with that in thyroid epithelial cell line (Figure 4b). The primary localization of LINC00475 in the cytoplasm of PTC cells was presented by subcellular fractionation assays (Figure 4c). The knockdown efficiency of sh-LINC00475#1/2 in PTC cells was examined by RT-qPCR, implying that the level of LINC00475 in PTC cells was strongly knocked down after transfection of sh-LINC00475#1/2 (Figure 4d). The binding area between LINC00475 and miR-376c-3p was searched on the LncBase website (Figure 4e). Luciferase reporter assays showed that LINC00475 silencing increased the luciferase activity of miR-376c-3p-Wt rather than that of miR-376c-3p-Mut, which confirmed the binding between LINC00475 and miR-376-3p at the predicted site (Figure 4f). As shown by RIP assays, ZCCHC12, miR-376c-3p, and LINC00475 largely coexisted in the Ago2 antibody. The enrichment of ZCCHC12 was the highest and that of miR-367c-3p was the lowest in PTC cells. The findings demonstrated that ZCCHC12, miR-376c-3p, and LINC00475 coexisted in the same RNA-induced silencing complex (Figure 4g). As presented by RT-qPCR and western blot, silenced LINC00475 upregulated miR-376c-3p expression while reducing mRNA and protein levels of ZCCHC12 in TPC1 and BCPAP cells (Figure 4h–j). Collectively, LINC00475 interacts with miR-376c-3p to upregulate the level of ZCCHC12.

### 3.5 LINC00475 promotes cell viability, proliferation, migration, and invasion by upregulating ZCCHC12 expression

**Figure 5:**
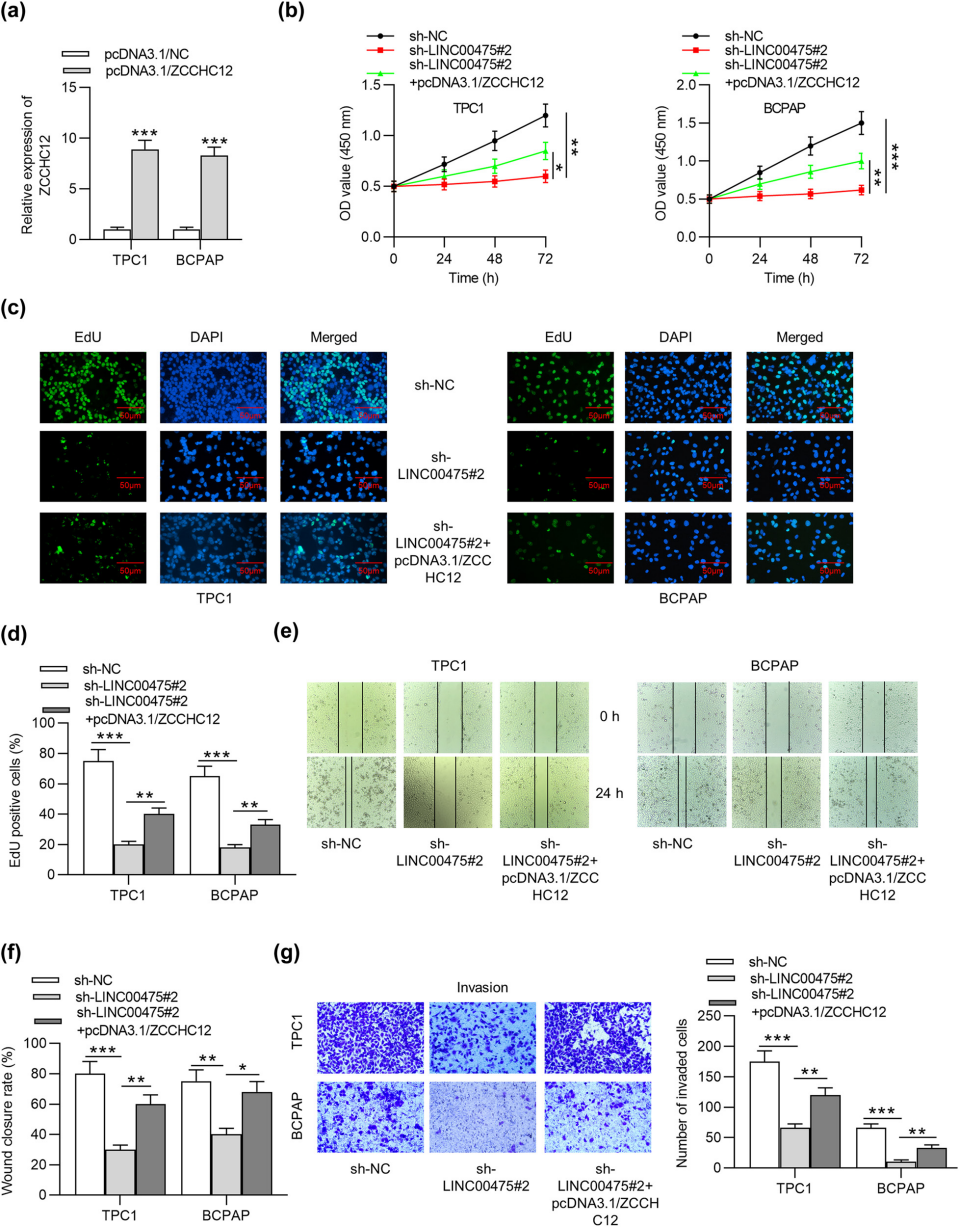
LINC00475 promotes PTC cell viability, proliferation, migration, and invasion by upregulating ZCCHC12 expression. (a) The expression level of ZCCHC12 after transfection with pcDNA3.1/NC and pcDNA3.1/ZCCHC12 in PTC cells was assessed by RT-qPCR analysis. (b–g) After the above transfection, CCK-8 assays were applied to evaluate cell viability, EdU assays were used to assess cell proliferation, wound healing assays were conducted to measure cell migration, and transwell assays were performed to detect cell invasion. *p < 0.05, **p < 0.01, ***p < 0.001.

Rescue experiments were designed to explore whether LINC00475 facilitates malignant behaviors of PTC cells by increasing ZCCHC12 expression. As presented in RT-qPCR, ZCCHC12 was strongly overexpressed after transfection of pcDNA3.1/ZCCHC12 in TPC1 and BCPAP cells (Figure 5a). CCK-8 and colony formation assays implied that the inhibitory effect of LINC00475 on cell viability and proliferation was partially reversed by ZCCHC12 overexpression (Figure 5b and c). Moreover, the migration and invasion of PTC cells were suppressed due to LINC00475 deficiency, and the suppressive effect was rescued by overexpressed ZCCHC12 as presented by wound healing assays and transwell assays (Figure 5d and e). In summary, LINC00475 promotes cell viability, proliferation, migration, and invasion by upregulating the ZCCHC12 level.

## 4 Discussion

PTC is a common subtype of thyroid carcinoma, accounting for more than 80% of all thyroid cancer cases [24,25]. In recent years, accumulating lncRNAs have been revealed to be implicated in PTC progression [14,15,20]. According to bioinformatics analysis, LINC00475 is highly expressed in thyroid carcinoma tissue samples (*n* = 510). Our study revealed that LINC00475 expression was upregulated in PTC cells. LINC00475 silencing suppressed PTC cell viability, proliferation, migration, and invasion. Overall, LINC00475 plays an oncogenic role in PTC progression.

Furthermore, LINC00475 was found to function as a ceRNA for miR-376c-3p in PTC. In this study, LINC00475 was verified to be primarily distributed in the cytoplasm of PTC cells. As ceRNAs are transcripts that regulate each other post-transcriptionally by competing for shared miRNAs [26], we confirmed that LINC00475 can function at a post-transcriptional level as a ceRNA. Previously, LINC00475 was reported to be mainly localized in both cytoplasm and nucleus of LN229 cells (glioma). Under hypoxia, nuclear LINC00475 is largely transcribed to the cytoplasm in LN229 cells and then it acts as a ceRNA to upregulate AGAP2 by binding to miR-449b-5p [27]. In our study, we only explored the relationship of LINC00475, miR-376c-3p, and ZCCHC12 in the cytoplasm and did not explore the regulatory role of nuclear LINC00475. We hypothesized that nuclear LINC00475 might also affect PTC cellular processes in another manner, which will be focused on in our future studies.

Mechanistically, lncRNAs interact with miRNAs to regulate the expression of miRNA targets by serving as a ceRNA [28]. miRNAs are small, highly conserved RNA molecules encoded in the genome of animals and plants, which can regulate the expression of genes by binding to the 3′-UTR of specific mRNAs [29]. We herein found that miR-376c-3p has a binding site for LINC00475. Based on previous studies, miR-376c-3p was involved in hepatocellular carcinoma, breast cancer, and PTC [30,31,32]. LINC01278 suppresses PTC development by binding to miR-376c-3p as a ceRNA to upregulate the expression of dynamin 3 [32]. In this study, miR-376c-3p expressed a low level in PTC cells, and its expression was negatively correlated with LINC00475 expression. The binding capacity between LINC00475 and miR-376c-3p has been verified. Moreover, LINC00475 functioned as a ceRNA against miR-376c-3p to antagonize the inhibitory effect of miR-376c-3p on ZCCHC12 expression in PTC.

ZCCHC12, also named Sizn1, is a metastasis-associated oncogene in PTC and has significant biological functions [22]. High ZCCHC12 expression in thyroid malignancy has been revealed by bioinformatics analysis and previous studies [22,23]. ZCCHC12 depletion suppresses the growth, migration, and invasion of PTC cells [22]. Mahmoudian and Forghanifard discovered that correlation between different cell signaling pathway-related genes, including ZCCHC12, may lead to the tumorigenesis of esophageal cancer [33]. Consistent with these views, our study found that ZCCHC12 was upregulated in PTC cells and the knockdown of ZCCHC12 inhibited PTC cell viability, proliferation, migration, and invasion. We also first discovered the ceRNA network of LINC00475/miR-376c-3p/ZCCHC12 in PTC. Furthermore, ZCCHC12 was reported to localize to promyelocytic leukemia protein nuclear bodies that are found in the nucleus as large ring-shaped protein complexes [34]. Another study reveals that ZCCHC12 is a nuclear protein to activate the transcriptional activities of activator protein 1 and cAMP response element-binding protein signaling [35]. We herein found that ZCCHC12 was mainly distributed in the cytoplasm of PTC cells. However, the nucleus localization of ZCCHC12 might also be important for its promoting effects on cellular behaviors of PTC cells as a fraction of LINC00475 is localized in the nucleus. More experiments will be conducted in the future to investigate more specific functions of LINC00475 and ZCCHC12 in PTC cells.

In summary, LINC00475 functions as a ceRNA against miR-376c-3p to antagonize the inhibitory effect of miR-376c-3p on ZCCHC12 expression, thereby promoting PTC cell proliferation, migration, and invasion. Our study may provide novel therapeutic targets of PTC. However, *in vivo* experiments were not included in this study, which will be conducted in our future exploration. More studies will be done to investigate the LINC00475/miR-376c-3p/ZCCHC12 pathway in PTC development.
