# Expression of concern “A ceRNA network mediated by LINC00475 in papillary thyroid carcinoma”

**DOI:** 10.1515/med-2025-9996

**Published:** 2025-04-01

**Authors:** Editors of Open Medicine

Expression of Concern: Yang Y, Hua W, Zeng M, Yu L, Zhang B, Wen L. A ceRNA network mediated by LINC00475 in papillary thyroid carcinoma. Open Medicine. 2022;17(1):22–33. https://doi.org/10.1515/med-2021-0389 by Editors of Open Medicine.

With this notice, the Editorial Board of Open Medicine would like to inform readers that Figures 2e, 5e and 2g, 5g were included in the aforementioned article by mistake. These incorrect figures raise questions about the integrity and rigor of the published research. Despite multiple attempts to contact the authors for clarification, no satisfactory response has been received.

The journal’s Editorial Board and the Publication Ethics Committee are investigating the article in accordance with the Committee on Publication Ethics Guidelines and De Gruyter Brill procedures. The Expression of Concern will be updated as soon as the investigation is complete.

